# The Effects of Volatile Organic Compounds (VOCs) on the Formation of Heterocyclic Amines (HAs) in Meat Patties, under Different Smoking Temperatures and Durations

**DOI:** 10.3390/foods11223687

**Published:** 2022-11-17

**Authors:** Xing Shen, Yang Chen, Jacob Ojobi Omedi, Emel Oz, Fatih Oz, Chunwang Xiao, Yijun Zhou, Jie Chen, Maomao Zeng

**Affiliations:** 1 College of Life and Environmental Sciences, Minzu University of China, Beijing 100081, China; 2 State Key Laboratory of Food Science and Technology, Jiangnan University, Wuxi 214122, China; 3 School of Food Science and Technology, Jiangnan University, Wuxi 214122, China; 4 Department of Food Engineering, Faculty of Agriculture, Ataturk University, Erzurum 25240, Turkey

**Keywords:** heterocyclic amines, smoking durations, smoking temperatures, volatile organic compounds, UPLC-MS/MS, meat patties

## Abstract

In this study, UPLC-MS/MS was used to study the effects of smoking duration and temperature on the formation of heterocyclic amines (HAs) in smoke-processed meat patties. Four kinds of free HAs—including F-7,8-DiMeIQx; F-MeAαC; F-Harman and F-Norharman—and six kinds of protein-bound HAs—including B-AαC; B-7,8-DiMeIQx; B-Glu-p-1; B-MeAαC; B-Harman and B-Norharman—were detected and quantified. Among the free HAs, we observed a 23-fold content increase (*p* < 0.05), from 0–4 h (at 0 h and 4 h they were 4.24 ng·g^−1^ and 98.33 ng·g^−1^, respectively), and the content of the free HAs decreased to 78.80 ng·g^−1^, at 5 h. At the same time, the free HAs content increased from 53.52 ng·g^−1^, at 50 °C, to 127.16 ng·g^−1^, at 60 °C, and then decreased continuously. The content of the free HAs was the highest at 60 °C. For the protein-bound HAs, their content was found to generally decrease with the increase in smoking duration and temperature. However, at 5 h, the content of protein-bound HAs slightly increased to 984.2 ng·g^−1^. Meanwhile, at 90 °C, it increased to 1643.53 ng·g^−1^. Additionally, a total of 16 volatile organic compounds (VOCs) were found in all of the meat samples, of which 10 VOCs (one acid, three aldehydes and seven phenols) were significantly related to the formation of free HAs. These findings showed that all the different types of HAs were produced under low-temperature processing, which provided scientific insights into the potential generation of HAs during meat smoking processes and could be used as a reference to minimize the risks of cancer related to the consumption of smoked meat products.

## 1. Introduction

Food smoking is one of the oldest food preservation techniques and belongs to a typical thermal processing food treatment method. During the smoking of meat products, the process flow generally involves curing, air drying, ripening and smoking [1]. With increasing consumer awareness, there has been increased research interest within the food industry on the quality and improvement of smoked meat [2,3]. To improve the quality of smoked meat, researchers have explored the significance of the smoking parameters—e.g., the chemical compositions of the smoke, and the smoking temperature and duration—in generating smoked foods, which are attributed to the diverse compounds present in smoked products due to the incomplete combustion of wood; the interactions between the smoked chemical components and the meat components; the effects of the heat generated by the wood combustion on the meat components (e.g., lipid, protein and carbohydrates); and the complex effects of smoking on the unique sensory and aroma qualities of meat products [4,5]. The published literature shows the different smoking conditions and smoked constituents that have been commonly explored to enhance the quality of smoked meat. For instance, phenolic compounds in smoked food have been found to be partly responsible for the flavor of meat and conferred antioxidant properties to smoked meat during storage, while carbonyl compounds have been associated with the attractive brown color formed on smoked meat’s surfaces [2]. However, the processing of smoked foods has been associated with the formation of and/or the production of hazardous substances such as polycyclic aromatic hydrocarbons and acrylamide [6,7]. With consumer safety and quality awareness in mind, little is known about the presence of heterocyclic amines (HAs) in smoked foods [8].

HAs are heterocyclic aromatic hydrocarbons with known carcinogenic and mutagenic properties. Presently, 30 HAs have been isolated and identified [9]. The International Agency for Research on Cancer (IARC) defined MeIQ, MeIQx, PhIP, Glu-P-1, AαC and MeAαC as class 2B carcinogens, and IQ as a class 2A carcinogen [10]. A high intake of HAs was reported to significantly increase the risks of cancer, particularly the incidence of colorectal cancer in a human case-control study [11]. A variety of HAs have been detected in several heat-processed meat products, such as sausages [12], grilled fish [13], smoked and roasted poultry [8], etc., indicating that humans are at risk of continuous exposure to HAs through their dietary intake. Ordinary thermal processing, such as hot air drying or baking, can cause water to migrate on food surfaces, enhance the migration of HAs’ precursors and increase water mobility during the drying effect in the smoking process, resulting in the accumulation and formation of HAs on food surfaces. However, the effects of smoking on the formation of HAs differs from other thermal processing processes. The constituents of the smoke during the smoking process have an additional influence on the carbonyl compounds of HAs’ intermediates and can lead to the introduction of phenolic compounds (HA inhibitors) [14]. Various phenolic compounds were detected in smoke fueled by jarrah, karri, marri, oats and pine, with their content varying greatly, based on the type of fuel used [15]. Recent studies on the formation mechanism have revealed that reactive carbonyls, such as phenylacetaldehyde, acrolein and crotonaldehyde, were intermediate products of HAs’ formation. An increase or decrease in these intermediates [16] can change the levels of HAs, which may significantly affect the Maillard reaction pathway, resulting in the uncertainty of HAs’ formation. Thus, studying the formation and accumulation of HAs in smoked meat might offer several practical significances.

Considering that exploring the influence of different smoking processing methods on the formation of HAs in smoked meat could provide a basis for food quality control in the smoking process stage—which could be adaptable in the industrialized production of smoked food—the objectives of this study were to analyze the formation of HAs during different meat smoking conditions, including different temperatures (50, 60, 70, 80 and 90 °C) and durations (1, 2, 3, 4 and 5 h), and to study the VOCs in meat patties under different processing conditions. The relationship between each VOC and HAs’ formation was ascertained.

## 2. Materials and Methods

### 2.1. Chemicals and Materials

HAs’ standards—including 2-amino-9H-pyrido[2,3-b]indole (AαC); 2-amino-3-methyl-9H-pyrido[2,3-b]indole (MeAαC); 1-methyl-9H-pyrido[3,4-b]indole (Harman); 9H-pyrido[3,4-b]indole (Norharman); 2-amino-3-methylimidazo[4,5-f]quinoline (IQ); 2-amino-3,4-dimethylimidazo[4,5-f]quinoline (MeIQ); 2-amino-1-methylimidazo[4,5-b]quinoline (IQ[4,5-b]); 2-amino-1-methyl-6-phenylimidazo[4,5-b]pyridine (PhIP); 2-amino-1,6-dimethylimidazo[4,5-b]pyridine (DMIP); 2-amino-1,5,6-trimethylimidazo[4,5-b]pyridine (1,5,6-TMIP); 2-amino-3-methyl-3H-imidazo[4,5-f]quinoxaline (IQx); 2-amino-3,8-dimethylimidazo[4,5-f]quinoxaline (MeIQx); 2-amino-3,4,8-trimethylimidazo[4,5-f]quinoxaline (4,8-DiMeIQx); 2-amino-3,7,8-trimethyl-3H-imidazo[4,5-f]quinoxaline (7,8-Di-MeIQx); 2-amino-3,4,7,8-tetramethyl-3H-imidazo[4,5-f]quinoxaline (4,7,8-TriMeIQx); 2-amino-5-phenylpyridine (Phe-P-1); and 2-amino-6-methyldipyrido[1,2-α:3′,2′-d]imidazole (Glu-P-1)—were purchased from Santa Cruz Biotechnology, Inc. (Santa Cruz, CA, USA). All standards had a purity level >99.9%. All 17 HAs were diluted with chromatographic grade methanol to a standard solution (125 mg·L^−1^). UPLC-MS/MS-grade methanol, acetonitrile and formic acids were obtained from Thermo Fisher Scientific (Waltham, MA, USA). Analytical-grade diatomaceous earth was purchased from Sinopharm Chemical Reagent Co., Ltd. (Shanghai, China). Oasis MCX cartridges (3 cc, 60 mg^−1^) were purchased from Waters (Shanghai, China). Pork and salt were purchased from a local supermarket (Wuxi, China).

### 2.2. Preparation of Meat Patties

Pork (tenderloin) was thoroughly ground using a meat mincer (BJRJ-12T, Jiaxing, China). The meat tendons were removed, followed by adding 1.5% NaCl, rolling the mixture for 1 h in a vacuum curing machine (Sunhow KA-6189A, Shenzhen, China) and pickling under vacuum refrigeration at 4 °C, for 18–20 h. Lastly, the minced meat (80 g) was pressed into 10 cm diameter meat patties, using a meat patty forming machine (Hualing ZF-100, Maanshan, China).

### 2.3. Smoking of Meat Patties

**Figure 1:**
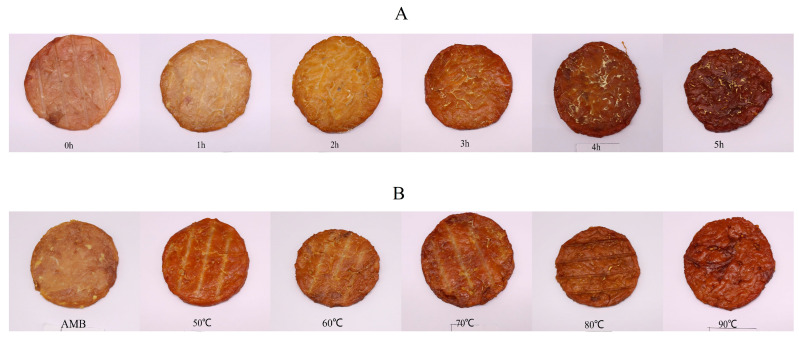
Smoked meat patties at different smoking durations (**A**) and temperatures (**B**). Group 1, in which the meat patties were smoked for 0, 1, 2, 3, 4 and 5 h, at 70 °C; and Group 2, in which the meat patties were smoked for 3 h, at ambient temperatures (AMB), 50, 60, 70, 80 and 90 °C.

The meat patties were dried for 0.5 h in the Self-Cooking Center of RATIONAL SCC 61 (Landsberg, Germany), whose oven was pre-heated to 70 °C. The measurement was conducted in triplicate. The meat patties were then naturally cooled at room temperature. After cooling, they were separated into the following two treatment groups: Group 1, in which the meat patties were smoked for 0, 1, 2, 3, 4 and 5 h, at 70 °C; and Group 2, in which the meat patties were smoked for 3 h at ambient temperatures (AMB), 50, 60, 70, 80 and 90 °C. As each sample took a long time to be smoked, it took 2–3 days to complete the total processing of each group. Therefore, the meat of the two groups was purchased separately. In all treatments, the smoke was produced by VarioSmoker 61-201 (Landsberg, Germany). VarioSmoker was used together with Self-Cooking Center. In the experiment, the weight of woodchips used for each fumigation was 120 g, and the VarioSmoker was put in the Self-Cooking Center, as shown in Figure S1. A total of 12 kinds (Figure 1) and 36 smoked meat patties were produced, with each treatment conducted in triplicate. After smoking, the meat patties were cooled at room temperature and weighed. Lastly, the smoked meat patties were crushed in a food processor, freeze-dried and stored at −20 °C for further analysis.

### 2.4. Analysis of Conventional Physicochemical Properties of Smoked Pork Patties

The surface color of the smoked meat patties was determined according to the method of Zhang et al. [17], but we made a slight change. An automatic colorimeter (Kangguang WB-2000IXA, Beijing, China) was used to measure the color of freeze-dried smoked meat patties. The instrument was calibrated using a standard whiteboard (X = 83.24, Y = 87.98, Z = 91.61). A standard D65 light source, with a 10° field of view, a Φ18 mm reflection measurement aperture and a Φ10 mm projection measurement aperture was also used. After calibration, the meat patty was tiled on the round measuring port for measurement. Then, the lightness (L*), redness (a*) and yellowness (b*) of the sample were recorded. Each sample was analyzed in triplicate.

The water activity (Aw, AQUALAB 4TEV, Jinan, China) and cooking loss were detected according to the method described by Zhang et al. [17].

The changes in meat pH (METTLER TOLEDO FE20, Shanghai, China) were determined using a previously described assay [18].

The texture properties of the pork patties were measured by TA. XT plus texture analyzer (Stable Micro Systems Ltd., Godalming, UK) was used according to the previously described methods, with some modifications [19]. A flat-bottomed cylindrical probe, P/36R (36 mm diameter), was used to perform texture profile analysis (TPA). The test conditions used were as follows: 3 mm·s^−1^ pre-test speed, 2 mm·s^−1^ test speed, 3 mm·s^−1^ post-test speed, 50% strain, 5 s dwell time interval and a trigger force of 5 g.

### 2.5. Determination of the VOCs in Smoked Pork Patties

The VOCs in the pork patties were identified by GC-MS (GCMS-QP2020NX, Shimadzu, Kyoto, Japan), according to the methods described by Rizzo et al. [20], with some modifications. Briefly, 2 g pork patty powder was added to 20 mL glass vials, with Teflon silicon septa lids. An internal standard of 20 µL of 2-methyl-3-heptanone (118.2 μg·L^−1^ in methanol) was added to each vial before SPME fiber exposure. The SPME fibers used were 1.0 cm in length with DVB/CAR/PDMS coating (Supelco, Bellefonte, PA, USA). Before exposure to the sample bottle, the SPME fiber was pre-heated at 270 °C, for 30 min, in the GC inlet. The sample bottles were equilibrated to 50 °C, for 30 min, during exposure. The samples were then manually injected at a depth of 5.0 cm into a split-less GC inlet, at 250 °C, onto an SH-WAX column (30 m × 0.25 mm, 0.25 µm; Shimadzu), with a constant flow rate of 0.8 mL·min^−1^ of helium. The condition of GC-MS was set as described by Rizzo et al. [20], with some modifications. The GC oven was initially set to 40 °C and held for 3 min. The temperature ramp began at a rate of 6 °C·min^−1^ to 100 °C, then accelerated to 10 °C·min^−1^ to 230 °C, which was held for 5 min. The purge time was set for 1 min. The MS transfer line was held at 250 °C, with the ion source held at 200 °C.

### 2.6. Sample Preparation for HAs

Free HAs in smoked meat were extracted and concentrated according to the method described by Zeng et al. [21], with some modifications. Protein-bound HAs were extracted according to the method described by Chen et al. [22]. Briefly, 3 g of smoked meat powder and 30 mL sodium hydroxide (1 mol·L^−1^) solution was added and homogenized (12,000 rpm, 1 min) (RayKol AH-30, Xiamen, China). Then, 13 g of diatomite was added to the homogenate and mixed, followed by adding and mixing 50 mL ethyl acetate and ultrasonic (40 KH_Z_, 250 W, SCIENTZ SB-5200, Ningbo, China) treatment, for 30 min. The extraction procedure was repeated twice, followed by centrifugation (10,000× *g*, 10 min) to obtain a supernatant, which was concentrated with nitrogen to 10 mL. Furthermore, the filtrate was collected and added to a 48 mL thick-walled, pressure-resistant bottle, followed by the addition of 40 mL of 6 mol·L^−1^ HCl to the bottle, bubbling nitrogen through for 1 min and heating at 110 °C, for 24 h. Next, they were centrifuged and the supernatant collected was diluted with ultrapure water to 100 mL, from which 10 mL was used for solid phase extraction (SPE).

### 2.7. SPE Procedure

For free HAs, Oasis MCX cartridges were activated in advance with 6 mL water, 6 mL methanol and 6 mL ethyl acetate, by automatic SPE (RayKol Fotector-04HT, Xiamen, China). For protein-bound HAs, Oasis MCX cartridges were activated in advance with 6 mL methanol, 6 mL water and 6 mL 0.1 mol·L^−1^ HCl. Ten milliliters (10 mL) of extract were added to the SPE column, the effluent was discarded and they were rinsed with 6 mL 0.1 M HCl and 6 mL methanol. The retained HAs were eluted with 6 mL methanol-ammonia mixture (19:1, *v*/*v*), at a flow rate of 0.6 mL·min^−1^ [22]. The eluted mixtures were evaporated to dryness under nitrogen at 50 °C, dissolved in 250 μL methanol and filtered through a 0.22 μm syringe filter for UPLC-MS/MS analysis. All tests were performed in triplicate.

### 2.8. UPLC-MS/MS Analysis

The HAs extracted from smoked meat were identified and quantified using a UPLC system, equipped with a triple quadrupole mass spectrometer (Waters, Milford, MA, USA). Acquity BEH C18 column (2.1 × 100 mm, particle size 1.6 μm, Waters) was used. The mobile phases used consisted of A: 100% acetonitrile and B: ultrapure water + 0.1% formic acid. The elution gradient at a 45 °C column temperature was 2% A (0–2 min), 20% A (12 min), 100% A (14 min), 2% (17 min) and 2% A (20 min) [23]. The flow rate was 0.3 mL·min^−1^ and the injection volume was 2 μL. The mass spectrometry detection was conducted in multiple-reaction monitoring (MRM) scan mode, under positive electrospray ionization (ESI), as described by Li et al. [24], with some modifications. The operating conditions of mass spectrometry were as follows: 100 °C ion source temperature; 400 °C dissolved gas temperature; 700 L·h^−1^ flow rate; 3.5 kV capillary voltage; 50 L·h^−1^ cone flow (nitrogen); 0.15 mL·min^−1^ flow rate of collision gas (argon); and 2-2000 Da scanning range. According to the peak shape and intensity of different treatments, the channel reaction, dwell time, cone voltage, collision energy and delay time of 17 HAs in the MRM mode of this study were optimized (Table S1).

### 2.9. Statistical Analysis

The UPLC-MS/MS results were analyzed using the Masslynx v4.1 software (Waters). Data were reported as means ± standard deviation and analyzed using the one-way analysis of variance to determine significant differences (*p* < 0.05) among the treatments. Statistical analysis was performed using the general linear model program of the Statistic software v9.0 (Analytical Software, Tallahassee, FL, USA). HAs’ data were imported into SIMCA 14.1 (Umetrics, Umea, Sweden) for principal component analysis (PCA). Spearman rank-order correlations were assessed using the Origin Pro 2021b software (Northampton, NC, USA). The Origin Pro 2021b and Anaconda Jupyter Notebook v6.4.8 (Austin, TX, USA) software were used for drawing.

## 3. Results and Discussion

### 3.1. Conventional Physicochemical Properties of Smoked Meat Patties

The physicochemical properties of the smoked meat patties are presented in Table 1. The results showed that increasing the smoking duration and temperature significantly decreased the L* and increased the a* value of the smoked meat. This was attributed to the melanoids that formed on the surface of the meat through the Maillard reaction pathway [17]. These observations were consistent with those reported in similar research studies [25]. The pH value of the pork patties that were smoked at different durations was significantly lower than the control sample (0 h), which was possibly due to the natural decrease in the pH value of the meat products during thermal processing and an increase in the meat’s exposure to acidic smoke metabolites due to the increased smoking time [26]. Moreover, the increased duration and temperature during the smoking led to significantly increased water processing loss and decreased water content in the meat patties. At the same time, the hardness, springiness and chewiness increased (*p* < 0.05) in all the pork meat patties, which were associated with the changes in the water, protein and lipids’ constituents. The heating of the meat was associated with the thermal denaturation of the proteins and a simultaneous change in the binding of the water and oxidation of the lipids, which were reflected by the changes in the gel properties [27]. These assertions were confirmed in an earlier study, where the heat treatment resulted in protein coagulation in the interstitial spaces, collagen and lipids, forming a gel; the denaturation and coagulation of the myofibrillar and sarcoplasmic proteins; and the shrinking of the intermuscular connective tissue [28]. Furthermore, similar observations were confirmed in a recent study, where an electronic nose, electronic tongue and HS-SPME/GC-MS were used to evaluate cooked chicken legs, which showed that the water content, water activity, pH value and L* value decreased from 71.26% to 65.23%, from 0.987 to 0.979, from 6.66 to 5.36 and from 61.84 to 52.34, respectively (*p* < 0.05) [29].

### 3.2. Effect of Smoking Duration and Temperature on the Formation of HAs

**Figure 2:**
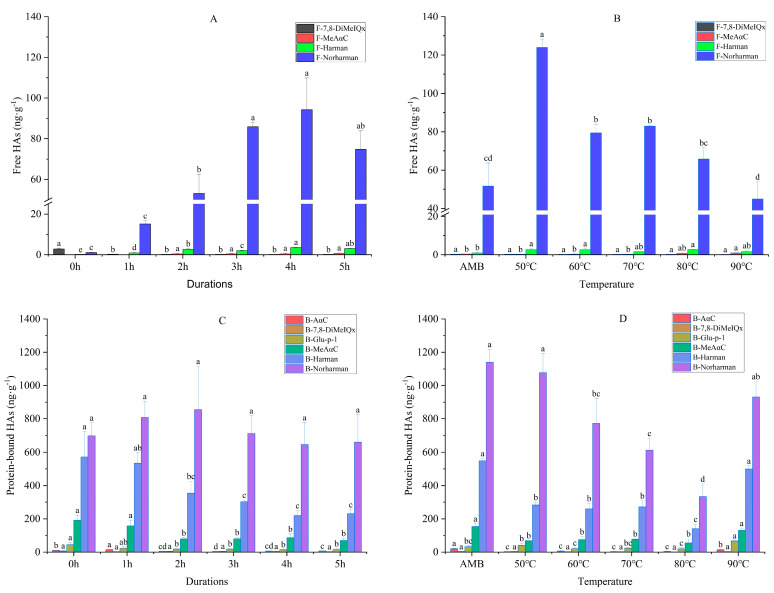
Changes in free HAs (**A**,**B**) and protein-bound HAs (**C**,**D**) in smoked meat patties, treated at different smoking durations and temperatures (ng·g^−1^). Data are presented as means ± standard deviations (*n* = 3). AMB represents ambient temperatures. Different letters in each treatment group represent significant differences at *p* < 0.05.

The effects of the smoking duration and temperature on the formation of HAs are presented in Figure 2. Four kinds of free HAs—namely F-7,8-DiMeIQx (0.15–2.89 ng·g^−1^); F-MeAαC (0–1 ng·g^−1^); F-Harman (0.2–3.6 ng·g^−1^) and F-Norharman (1.15–123.99 ng·g^−1^)—were detected in the smoked meat patties (Figure 2A,B). The results showed that the content of free HAs initially increased, then decreased with the increase in the smoking duration, reaching a maximum value of 98.33 ng·g^−1^, at 4 h, which was 23 times the total content, compared with the non-smoked meat (0 h) (4.24 ng·g^−1^). During the smoking and heating process, water was gradually lost and acted as a reaction and transmission medium for several precursor molecules on the surface of the meat patties. On the surface, the precursors were exposed to higher temperatures, resulting in the formation of HAs [30]. Compared with AMB, the total amount of free HAs decreased with an increase in the temperature, from 127.16 ng·g^−1^ to 47.82 ng·g^−1^. Among the four kinds of HAs, the content of F-Norharman was the highest, while the content of the other HAs changed little with the increase in the temperature. The amount of total free HAs decreased gradually, primarily due to a decrease in F-Norharman. A similar trend was observed with the formation of HAs in trout fillets that were cooked by sous-vide, under different cooking temperature conditions [31]. We hypothesized that the content of HAs could be explained by the rate of the thermal decomposition of some HAs being faster than their formation with an increase in the smoking temperature and time [32], which were concordant with the findings of Xi et al. [25], who reported that the rate of HAs’ formation significantly decreased with an increased temperature and time at some nodes. Furthermore, six kinds of protein-bound HAs—including B-AαC (2.53–20.83 ng·g^−1^); B-7,8-DiMeIQx (1.33–5.63 ng·g^−1^); B-Glu-p-1 (14.55–66.65 ng·g^−1^); B-MeAαC (54.37–191.3 ng·g^−1^); B-Harman (139.57–571.53 ng·g^−1^) and B-Norharman (333–1140 ng·g^−1^)—were detected in the smoked meat patties (Figure 2C,D). With an increase in the smoking duration, the content of B-Norharman initially increased slowly, and then decreased (600–800 ng·g^−1^), while the content of B-Harman decreased gradually (Figure 2C). At the same time, the content of B-Harman and B-Norharman first decreased, then increased with an increase in the smoking temperature (Figure 2D). The contents of the other four kinds of HAs were low, with no obvious change observed with the changes in the smoking duration and temperature. These observations were attributed to the effects of the temperature on the increased rate of the sample’s dehydration. In addition, the Maillard reaction and a series of complex reactions could have affected the formation of HAs. Harman and Norharman are the main HAs that are widely found in high contents in animal- [33] and plant-based [34] food products. This is due to Harman and Norharman being endogenous components—which are formed in natural mild conditions [35], and which could be detected in raw meat [36]—and their content may increase continuously with an increase in smoking temperature or time [25], until their degradation temperatures are reached [37]. In this study, protein-bound HAs were mainly formed in two ways: the bonding of free HAs with protein, or the involvement of amino acid residues on protein side chains in reactions that generated protein-bound HAs. In addition to HAs forming directly as a result of smoking, the changes in meat components, such as tryptophan and glutamate [38], and the effects of the smoke [39] on the protein and oil during smoking are related to free and protein-bound HAs. Generally, it is considered that HAs are produced at high temperatures; however, some HAs have been found to accumulate significantly at low temperatures. For instance, in an earlier study, the total content of HAs—based on AαC, Phe-P-1, DMIP, Harman and Norharman—was found to increase up to 1.41 μg·g^−1^ in raw sausage that was dried at 50 °C, for 10 min [40]. Furthermore, Norharman was produced in temperatures as low as 40 °C [41]. These findings imply that smoked meat products that are produced at relatively low temperatures might contain HAs. For quality control, it is, therefore, very important to control the production of HAs to help minimize the risk of cancer.

### 3.3. The VOCs of the Smoked Pork Patties

**Figure 3:**
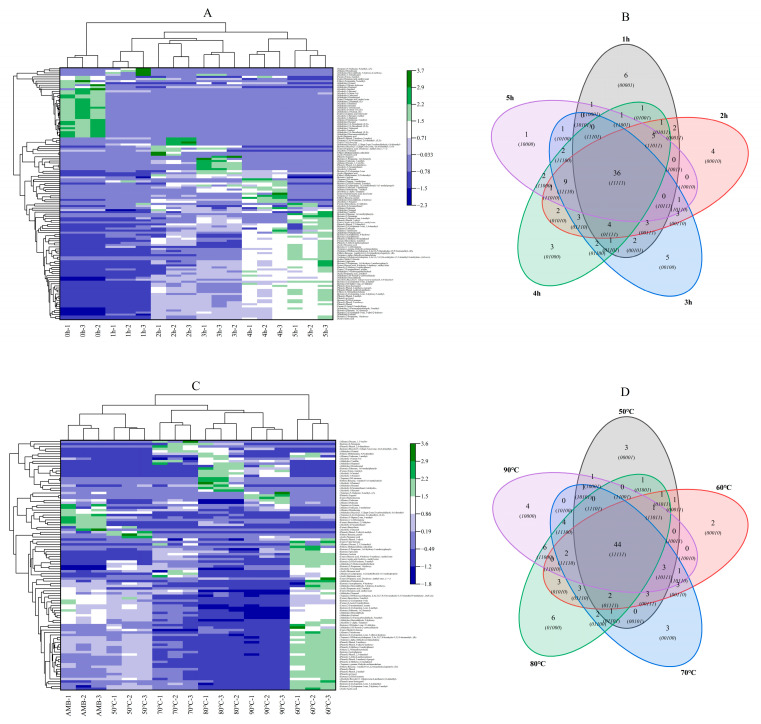
Cluster analysis diagram of VOCs in smoked pork patties at different durations (**A**) and temperatures (**C**). Wayne diagram of VOCs in smoked pork patties at different durations (**B**) and temperatures (**D**). AMB represents ambient temperatures.

There were 52 kinds of VOCs in the control sample of pork patties (0 h). An increase in the smoking duration from 1 h to 5 h was associated with the 63, 74, 72, 73 and 60 kinds of VOCs contained in the smoked pork patties, respectively (Figure 3). Zhenba bacon had the lowest VOC content when fresh. The type and content of VOCs increased with an increase in the treatment duration, resulting in a large number of phenolic substances [4]. There were 36 kinds of common VOCs and three kinds of VOCs, which included 10 ketones, 9 phenols and 5 aldehydes, which accounted for the main VOC. At the same time, compared with the 66 kinds of VOCs under AMB, an increase in smoking temperature from 50 °C to 90 °C resulted in 62, 69, 72, 76 and 67 kinds of VOCs, respectively, in the smoked pork patties. Among them, 44 kinds of VOCs were common at all temperatures. Similar to the duration gradient, ketones (11 kinds), phenols (11 kinds) and aldehydes (five kinds) were the main VOCs. Phenolic substances were mainly produced by a gradual decomposition of lignin and were unique volatile flavor substances in smoking [42]. It was mainly reflected in the generation of VOCs during the heating and decomposition of smoking substances, as well as phenolic substances formed after smoking [2]. Harman and Norharman are generally considered the products of protein pyrolysis, while aldehydes are reported to be the intermediates of β-carbolines HAs. Therefore, the presence of aldehydes in smoking can be regarded as the precursors that promote the formation of nonpolar HAs [43]. The VOCs in heated meat rise via major reaction mechanisms such as the Maillard reaction and lipid oxidation. A cluster analysis showed that the repeatability between the smoked pork patties was good, regardless of the duration or temperature of smoking. The six smoking durations were divided into three categories (Figure 3A), as follows: (i) 0 h; (ii) 1, 2 and 3 h; and (iii) 4 and 5 h. Similarly, the different smoking temperatures (Figure 3C) were divided into the following three categories: (i) AMB and 50 °C; (ii) 70, 80 and 90 °C; and (iii) 60 °C. Generally, the difference between the control (0 h) and other treatments was large, mainly because the control was not smoked and the VOCs were mainly the odor substance of the meat. The differences between the meat patties that were smoked for 1, 2 and 3 h and those that were smoked for 4 and 5 h may have arisen because the ingredients of the VOCs needed time to penetrate into the meat patties, and they then reacted with the ingredients of the meat patties. At the same time, the VOCs with a higher content in the control patties than in the smoked meat patties were mainly aldehydes and alcohols. With an increase in the smoking duration, the content of ketones, terpenes and phenols accumulated and increased in the meat patties. However, when the temperature was above 70 °C, the difference in the VOCs in the smoked meat was not obvious.

### 3.4. Correlation between VOCs and HAs in Meat Patties at Different Durations and Temperatures

**Figure 4:**
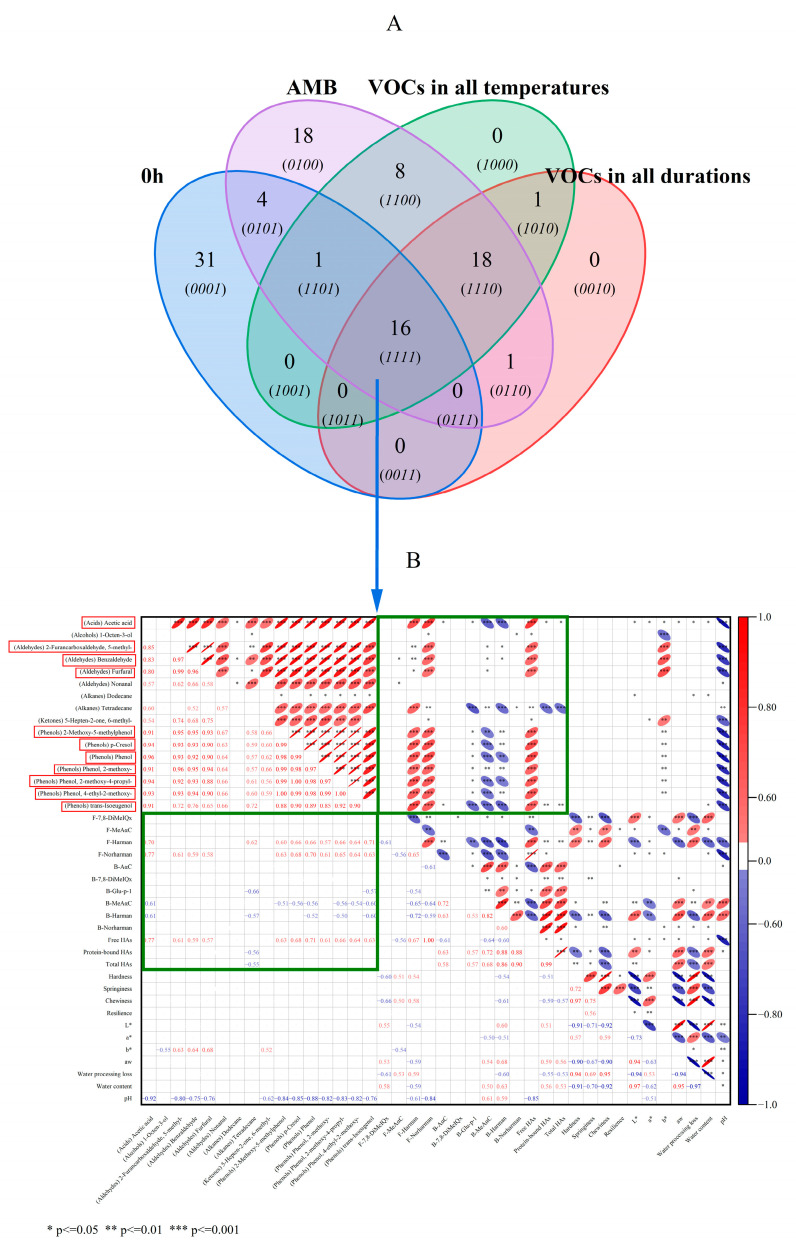
Wayne diagram of VOCs in non-smoked meat patties, at all durations and all temperatures of smoked meat patties (**A**). Correlation analysis of VOCs in all processed meat patties, HAs and other physical and chemical indexes (**B**). AMB represents ambient temperatures.

The Wayne mapping showed that there were 16 VOCs, including one acid, one alcohol, four aldehydes, two alkanes, one ketone and seven phenols, under different durations and temperatures (Figure 4A)—specifically comprising acetic acid; 1-Octen-3-ol; 2-Furancarboxaldehyde,5-methyl-; Benzaldehyde; Furfural Nonanal; Dodecane; Tetradecane; 5-Hepten-2-one,6-methyl-; 2-Methoxy-5-methylphenol; p-Cresol; Phenol; Phenol,2-methoxy-; Phenol,2-methoxy-4-propyl-; Phenol,4-ethyl-2-methoxy-; and trans-Isoeugenol. The proportion of phenol was very high. Different methods were used to distinguish the VOCs in the dry-cured hams, under different processing methods. The content of phenols, aromatic hydrocarbons and acids found in the smoked–cured, dry-cured hams was high and demonstrated the characteristics of smoking aroma [44]. A total of 27 VOCs with odor activity were identified as the key aroma compounds of smoked, cooked pork loin. The highest flavor dilution factor was 2-methoxyphenol; 2-methoxy-4-(propyl-2-enyl) phenol; and 2-methoxy-4-E)-(propyl-1-en-1-yl) phenol [45]. Among them, seven kinds of phenols were positively correlated with the free HAs (rs = 0.61–0.71, *p* ≤ 0.001), including five methoxyphenols. In principle, this confirmed that a darker color was associated with a higher content of HAs, which might be related to some phenolic compounds in smoking that promoted the formation of HAs. For instance, smoking-associated carbonyl compounds might provide intermediate compounds that promote the formation of HAs [11]. Mixed phenolic compounds have different effects on single HAs, some of which can effectively reduce their level, while others can effectively improve them [46]. It was also reported that the inhibitory activity of phenols on the formation of HAs was, at least partially, related to the content of phenols [47]. Among them, the obvious changes were as follows: (i) the content of 2-Methoxy-5-methylphenol, which increased from 9.13 μg·g^−1^, at 0 h, to 2935.41 μg·g^−1^, at 5 h; and (ii) Phenol,2-methoxy-, which increased from 11.72 μg·g^−1^, at 0 h, to 2789.87 μg·g^−1^, at 5 h. The content increased by 322 and 238 times, respectively, and gradually increased with the increase in the smoking duration (Figure S2A). With the gradual increase in the temperature, 2-Methoxy-5-methylphenol increased from 2367.75 μg·g^−1^, at AMB, to 4305.16 μg·g^−1^, at 60 °C, and then rapidly decreased to 642.58 μg·g^−1^, at 90 °C. Phenol,2-methoxy- followed the same trend, increasing from 2377.83 μg·g^−1^, at AMB, to 3393.7 μg·g^−1^, at 60 °C, and then dropping rapidly to only 803.27 μg·g^−1^, at 90 °C (Figure S2B). All of these changes were consistent with the results observed after the cluster analysis (Figure 3C). At 60 °C, the content of phenols was the highest and the effect was the most significant. The possible reason was that the pine woodchips enhanced the dealkylation and de-methoxylation of methoxyphenol with a rise in the smoking temperature [48]. At the same time, the acetic acid; 2-Furancarboxaldehyde,5-methyl-; Benzaldehyde; and Furfural were also positively correlated with the free HAs, with correlation coefficients (rs) of 0.77, 0.61, 0.59 and 0.57, respectively (*p* ≤ 0.001). Furthermore, 2-Methoxy-5-methylphenol; p-Cresol; Phenol; Phenol,2-methoxy-; Phenol,2-methoxy-4-propyl-; Phenol,4-ethyl-2-methoxy-; and trans-Isoeugenol showed a strong positive correlation with the free HAs, with correlation coefficients of 0.63, 0.68, 0.71, 0.61, 0.66, 0.64 and 0.57, respectively (*p* ≤ 0.001) (Figure 4B). However, different concentrations of phenols had quite different or opposite effects on the formation of HAs [49]. The possible reason was the different content of phenol. In roast beef patties, the addition of 0.05% and 0.10% chlorogenic acid significantly promoted the formation of PhIP (*p* < 0.05). Among them, tetradecane deserved special attention as it had a positive correlation with F-Harman (rs = 0.62, *p* ≤ 0.001) and a strong negative correlation with B-Glu-P-1 and B-Harman (rs = −0.66 and −0.57, *p* ≤ 0.001). In general, the smoking process significantly promoted the formation of HAs in meat products, due to the involvement of the smoking ingredients in producing HAs in meat products [50].

### 3.5. PCA Analysis of HAs, VOCs and Other Indexes in Smoked Meat Patties at Different Smoking Durations and Temperatures

**Figure 5:**
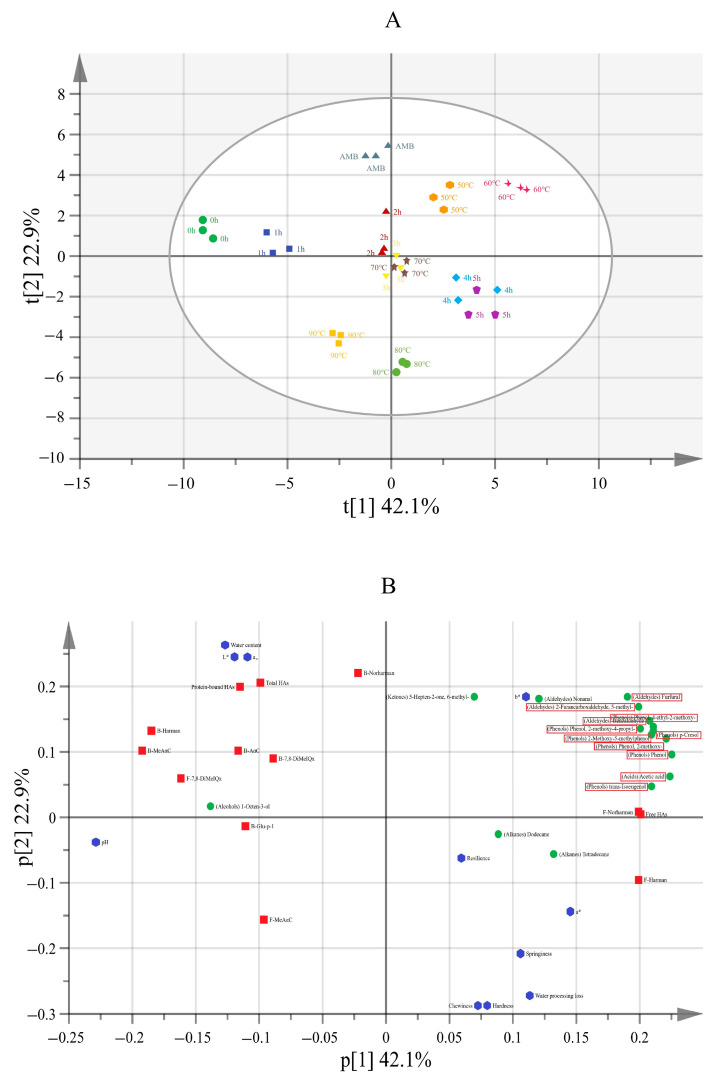
Scores (**A**) and loadings (**B**) scatter plot of PCA analysis of smoked meat, treated under different smoking durations and temperatures. AMB represents ambient temperatures.

Two principal components were extracted, representing 65% total variance, with component one (PC1) and component two (PC2) contributing 42.1 and 22.9% of the variance, respectively. As shown in the score scatter plot (Figure 5A), clear clusters and separations among the samples were seen. For instance, as the duration increased, the samples were scattered from left to right along the X axis, whereas, with the increase in temperature, the samples were dispersed from the top to the bottom along the Y axis. Unsmoked meat (0 h, control) was positioned at the far left of the first quadrant, while those after 4 and 5 h of smoking were located in the third quadrant. AMB was at the top, while those heated at 50 and 60 °C were located in the second quadrant, and those heated at 90 °C were located in the lower left corner. The results showed that the smoking duration and the temperature significantly influenced the content of HAs in the smoked meat patties. Furthermore, the loading scatter plot (Figure 5B) showed that, except for B-Glu-p-1, the other five types of protein-bound HAs were located in the first quadrant—similar to the positions of 0 h, 1 h and AMB in the score scatter plot—implying that the content of the protein-bound HAs was higher in the unsmoked meat patties when the smoking was short and at low smoking temperatures. The positions of F-Harman and F-Norharman on the loading scatter plot were similar to those at 4 h and 5 h on the score scatter plot. An increase in the smoking duration significantly increased the content of free HAs, compared with a short smoking duration. It was particularly interesting that VOCs positively correlated with free HAs—they were concentrated in the second quadrant and were in a similar position at 60 °C on the score scatter plot, indicating that the content of VOCs was high at 60 °C. Yang et al. [12] found that the total amount of free HAs in 60 °C smoked sausage was 5.49 ng·g^−1^ (*p* < 0.05), in which the Harman content was 0.85 ng·g^−1^ and the Norharman content was 5.81 ng·g^−1^. The TPA indexes were all concentrated in the third quadrant, with the same quadrant of 80 °C samples representing a higher processing temperature or 4 h and 5 h of a longer processing duration in the loading scatter plot. An increase in the smoking duration and temperature made the texture of the smoked meat patties hard and significantly deteriorated their chewiness, elasticity and resilience.

## 4. Conclusions

A low temperature for meat processing significantly increased the generation of various kinds of HAs. During the smoking of meat, some components may induce the production of HAs in the smoked meat. The 16 VOCs that existed under different conditions deserved special attention. In this study, the concentration of various HAs varied with the changes in the smoking durations and temperatures. In particular, VOCs with a high correlation with free HAs were concentrated in the second quadrant, corresponding to the position of 60 °C in the scores scatter plot of PCA. This also coincided with the situation that 60 °C was the most different from the other smoking temperature conditions in the cluster analysis. Our results showed that the smoking process significantly affected the formation of HAs in meat products. Therefore, the smoking of meat should be carefully controlled to minimize cancer risks.

## Figures and Tables

**Table 1:** The conventional physical and chemical indicators of smoked pork patties, with different smoking durations and temperatures.

		Hardness (g)	Springiness (%)	Chewiness (g)	Resilience (%)	L*	a*	b*	a_w_	Water Processing Loss (g·100 g^−1^)	Water Content (g·100 g^−1^)	pH
Duration	0 h	863.21 ± 104.96 d	0.63 ± 0.02 b	357.23 ± 73.54 c	0.23 ± 0.01 b	69 ± 0.93 a	6.7 ± 0.14 c	21.25 ± 0.56 c	0.97 ± 0 a	29.84 ± 0.35 f	66.13 ± 0.1 a	5.82 ± 0.01 a
	1 h	2425.06 ± 274.87 c	0.74 ± 0.05 a	1098.44 ± 127.31 bc	0.25 ± 0.01 ab	65.17 ± 0.38 b	8.31 ± 1.15 c	29.28 ± 1.16 ab	0.97 ± 0 b	32.97 ± 0.46 e	63.75 ± 0.45 b	5.66 ± 0.03 b
	2 h	3362.42 ± 469.36 bc	0.71 ± 0.01 ab	1474.3 ± 218.88 b	0.22 ± 0.01 b	55.74 ± 0.25 c	11.39 ± 0.91 b	28.23 ± 0.67 ab	0.96 ± 0 b	39.63 ± 0.45 d	59.26 ± 0.32 c	5.49 ± 0.01 c
	3 h	4607.45 ± 533.33 b	0.72 ± 0.04 ab	2022.51 ± 186.17 b	0.22 ± 0.01 b	50.42 ± 1.15 d	13.11 ± 1.2 ab	27.09 ± 0.86 b	0.96 ± 0 c	43.2 ± 0.73 c	53.34 ± 0.6 d	5.38 ± 0.01 d
	4 h	8719.97 ± 830.69 a	0.78 ± 0.01 a	4866.18 ± 645.94 a	0.27 ± 0.01 a	44.8 ± 1.15 e	15.26 ± 0.71 a	30.02 ± 1.24 a	0.95 ± 0 d	47.84 ± 0.64 b	50.58 ± 0.53 e	5.28 ± 0.03 e
	5 h	8689.31 ± 331.12 a	0.79 ± 0.06 a	4555.57 ± 509.58 a	0.24 ± 0.02 ab	31.04 ± 2.27 f	14.35 ± 0.22 a	23.55 ± 0.94 c	0.92 ± 0 e	57.31 ± 0.6 a	44.84 ± 0.19 f	5.23 ± 0.02 e
Temperature	AMB	1679.25 ± 106.28 c	0.7 ± 0.02 b	741.84 ± 100.64 b	0.26 ± 0.02 a	57.3 ± 0.34 a	14.15 ± 0.7 c	35.55 ± 1.17 a	0.97 ± 0 a	30.17 ± 0.24 e	62.96 ± 0.23 a	5.42 ± 0.02 a
	50 °C	2588.34 ± 127.49 bc	0.7 ± 0.05 b	1172.55 ± 60.61 b	0.24 ± 0.02 a	54.41 ± 0.72 b	16.21 ± 0.46 bc	35.81 ± 1.97 a	0.96 ± 0 b	34.97 ± 0.75 d	60.45 ± 0.51 b	5.12 ± 0.01 c
	60 °C	2453.55 ± 183.92 bc	0.75 ± 0.03 ab	1215.88 ± 68.46 b	0.26 ± 0 a	51.63 ± 0.48 c	16.17 ± 0.92 bc	33.92 ± 0.89 ab	0.96 ± 0 c	37.24 ± 0.46 c	56.84 ± 0.16 c	5.03 ± 0.01 d
	70 °C	4163.07 ± 486.14 b	0.69 ± 0.02 b	1760.74 ± 205.96 b	0.23 ± 0.02 a	48.06 ± 1.87 d	15.75 ± 0.38 c	32.33 ± 0.76 b	0.95 ± 0 d	42.29 ± 0.62 b	53.16 ± 0.22 d	5.38 ± 0.01 b
	80 °C	8969.06 ± 1306.85 a	0.8 ± 0.02 a	5803.19 ± 798.57 a	0.26 ± 0.01 a	33.87 ± 0.93 e	20.93 ± 2.04 a	28.13 ± 0.82 c	0.94 ± 0 e	49.19 ± 1.33 a	47.69 ± 0.27 e	5.43 ± 0.02 a
	90 °C	10288.56 ± 791.83 a	0.81 ± 0.04 a	5602.76 ± 815.01 a	0.27 ± 0 a	27.77 ± 0.89 f	18.82 ± 0.54 ab	25.76 ± 0.95 c	0.94 ± 0 e	51.15 ± 0.5 a	46.46 ± 0.57 f	5.43 ± 0.02 a

Group 1: the meat patties were smoked for 0, 1, 2, 3, 4 and 5 h at 70 °C. Group 2: the meat patties were smoked for 3 h, at ambient temperatures (AMB), 50, 60, 70, 80 and 90 °C. Data are presented as means ± standard deviations (*n* = 3). Different letters in each treatment group represented significant differences at *p* < 0.05.

## Data Availability

The results obtained for all experiments performed are shown in the manuscript and Supplementary Materials, the raw data will be provided upon request.

## Supplementary Materials

The following supporting information can be downloaded at, https://www.mdpi.com/article/10.3390/foods11223687/s1: Figure S1—schematic diagram of smoke experiment device; Figure S2—VOC content (ng·g^−1^) of smoked meat patties at different durations (A) and temperatures (B); And Table S1—mass spectrometric parameters of HAs in MRM mode of UPLC-MS/MS.
