# Supplementary material for: The Effects of Volatile Organic Compounds (VOCs) on the Formation of Heterocyclic Amines (HAs) in Meat Patties, under Different Smoking Temperatures and Durations
Source: Foods. 2022 Nov 17;11(22):3687. doi: 10.3390/foods11223687 (PMC9689661; doi:10.3390/foods11223687)
Supplement: Supplementary file 1 [file foods-11-03687-s001.zip › foods-2026394-supplementary.pdf]

Supporting Information

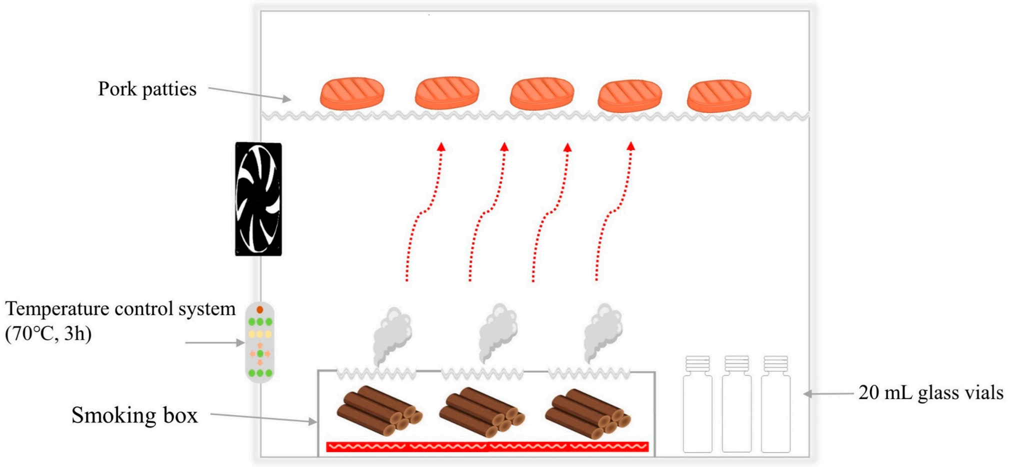

Figure S1. Schematic diagram of smoke experiment device.

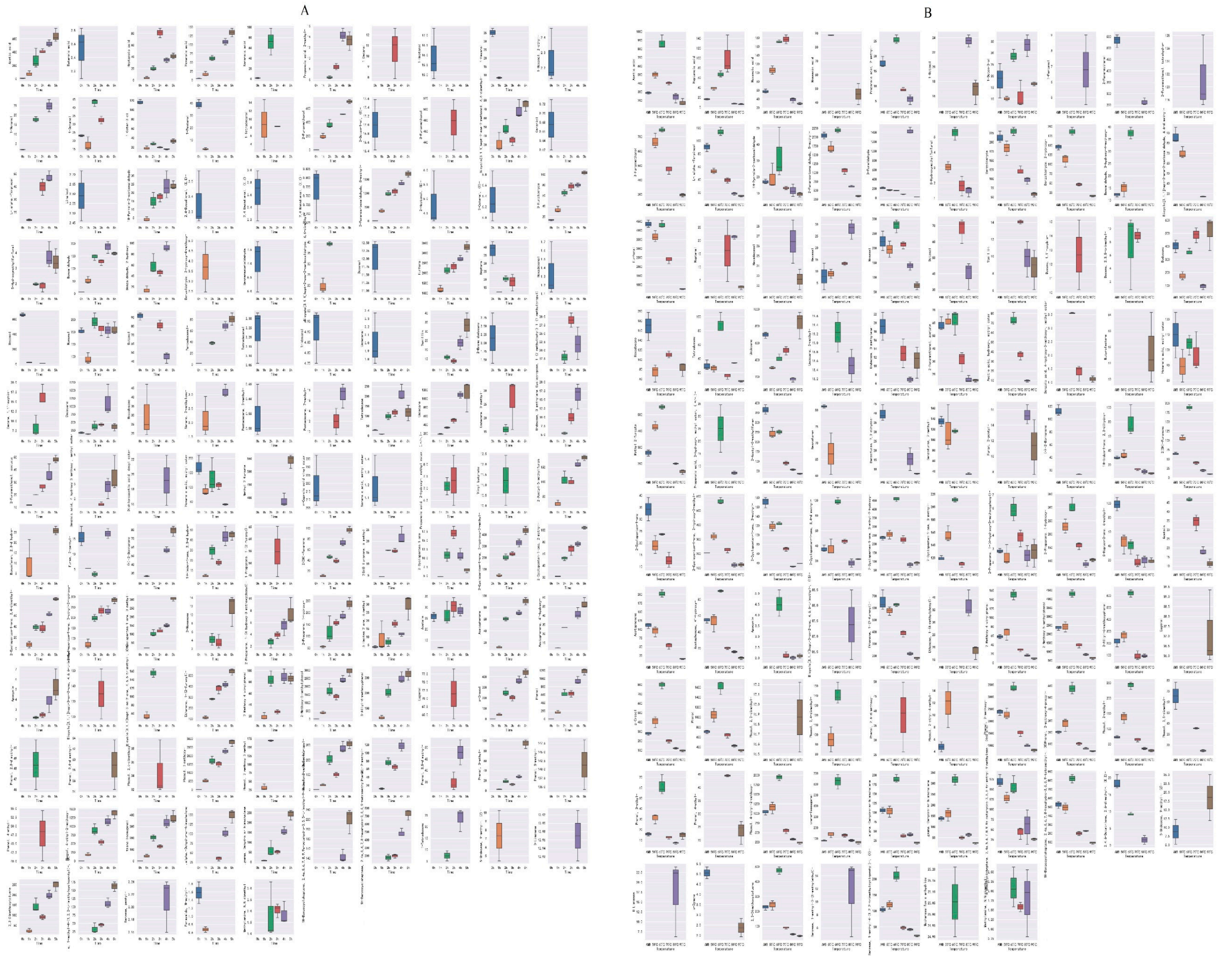

Figure S1. VOCs content (ng·g^{-1}) of smoked meat patties at different durations (A) and temperatures (B).

Table S1. Mass spectrometric parameters of HAs in MRM mode of UPLC-MS/MS.

	compound	channel reaction	dwell time (s)	cone voltage (V)	collision energy (eV)	delay time (s)
1	AαC	183.00 > 140.00	0.15	30	30	0.1
2	IQ	199.00 > 130.00	0.15	30	40	0.02
3	MeIQx	214.00 > 131.00	0.15	30	40	0.02
4	PhIP	225.00 > 210.00	0.15	30	30	0.02
5	7,8-DiMeIQx	228.00 > 213.00	0.15	30	25	0.02
6	DMIP	163.00 > 148.00	0.15	30	25	0.1
7	Norharman	169.00 > 115.00	0.15	30	30	0.02
8	Phe-P-1	171.00 > 127.00	0.15	30	30	0.02
9	1,5,6-TMIP	177.00 > 162.00	0.15	30	25	0.02
10	Harman	183.00 > 115.00	0.15	30	30	0.02
11	MeAαC	198.00 > 181.00	0.15	30	25	0.02
12	Glu-P-1	199.00 > 145.00	0.15	30	30	0.02
13	IQx	200.00 > 185.00	0.15	30	25	0.02
14	IQ[4,5-*b*]	199.00 > 115.00	0.15	30	40	0.1
15	MeIQ	213.00 > 198.00	0.15	30	25	0.02
16	4,8-DiMeIQx	228.00 > 212.00	0.15	30	30	0.02
17	4,7,8-TriMeIQx	242.00 > 227.00	0.15	30	30	0.02
